# *Borrelia puertoricensis* in opossums (*Didelphis marsupialis*) from Colombia

**DOI:** 10.1186/s13071-023-06016-4

**Published:** 2023-12-04

**Authors:** Yesica López, Álvaro A. Faccini-Martínez, Sebastián Muñoz-Leal, Verónica Contreras, Alfonso Calderón, Ricardo Rivero, Marina Muñoz, Juan David Ramírez, Salim Mattar

**Affiliations:** 1 Instituto de Investigaciones Biológicas del Trópico, Universidad de Córdoba, Córdoba, Colombia; 2 Servicio de Infectología, Hospital Militar Central, Bogotá, Colombia; 3 Servicios y Asesorías en Infectología - SAI, Bogotá, Colombia; 4 Departamento de Ciencia Animal, Facultad de Ciencias Veterinarias, Universidad de Concepción, Chillán, Chile; 5 Centro de Investigaciones en Microbiología y Biotecnología-UR (CIMBIUR), Facultad de Ciencias Naturales, Universidad del Rosario, Bogotá, Colombia; 6 Molecular Microbiology Laboratory, Department of Pathology, Molecular and Cell-Based Medicine, Icahn School of Medicine at Mount Sinai, New York City, NY USA; 7 Facultad de Medicina, Universidad Militar Nueva Granada, Bogotá, Colombia; 8 Paul G. Allen School for Global Health, Washington State University, Pullman, WA USA

**Keywords:** Opossums, *Borrelia puertoricensis*, Tick-borne spirochetes, Colombia

## Abstract

**Background:**

The genus *Borrelia* comprises pathogenic species of bacteria that pose a significant risk to public health. *Borrelia* spp. are emerging or reemerging infectious agents worldwide with complex transmission cycles, and many species use rodents as vertebrate reservoir hosts. Spirochetes morphologically compatible with *Borrelia* have been recurrently observed in opossums; however, there is currently a lack of genetic evidence confirming infection or supporting that these marsupials are hosts of *Borrelia* spirochetes.

**Methods:**

During 2017, 53 serum samples of *Didelphis marsupialis* from the municipality of Colosó (department of Sucre, Colombia) were collected and allocated in a serum bank. DNA extracted from the serum samples was submitted to a *Borrelia* genus-specific real-time PCR targeting the 16S rRNA gene. Positive samples were subsequently derived from semi-nested PCR protocols to obtain large fragments of the 16S rRNA and *flaB* genes. Obtained amplicons were subjected to Sanger sequencing. One positive sample was randomly selected for next-generation sequencing (NGS). Obtained reads were mapped to genomes of *Borrelia* spp. and sequences of two genes used in a multilocus sequence typing scheme retrieved for taxonomic assignment and phylogenetic analyses.

**Results:**

Overall, 18.8% (10/53) of the samples were positive by qPCR. Of them, 80% (8/10) and 60% (6/10) were positive for the 16S rRNA and *flaB* genes after semi-nested PCRs, respectively. Bioinformatic analysis of one sample sequenced with NGS yielded 22 reads of genus *Borrelia* with different sizes. Two housekeeping genes, *rplB* and *pyrG*, were recovered. Nucleotide pairwise comparisons and phylogenetic analyses of 16S rRNA, *flaB, rplB* and *pyrG* genes showed that the *Borrelia* sp. found in opossums from Colosó corresponded to *Borrelia puertoricensis*.

**Conclusions:**

We describe the first molecular evidence to our knowledge of *B. puertoricensis* in Colombia, specifically in opossums, and the first detection of this spirochete in a vertebrate host since its isolation from *Ornithodoros puertoricensis* in Panama. This detection is also relevant because of the epidemiological importance of opossums as reservoirs of zoonotic diseases to humans.

**Graphical abstract:**

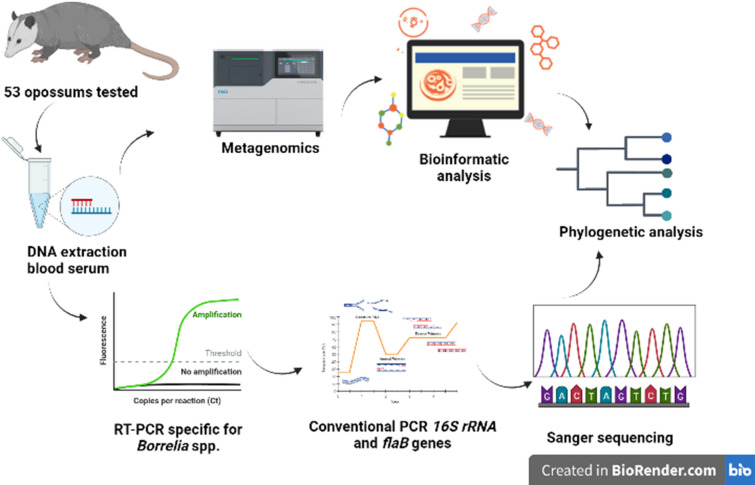

**Supplementary Information:**

The online version contains supplementary material available at 10.1186/s13071-023-06016-4.

## Background

Bacteria of the genus *Borrelia* are agents of emerging and re-emerging infectious diseases of domestic animals and humans and pose a threat to public health [1]. The main vectors of *Borrelia* bacteria are hard ticks of genus *Amblyomma*, *Ixodes* and *Rhipicephalus*; soft ticks of genus *Argas* and *Ornithodoros*; and the human clothing louse *Pediculus humanus humanus* [2].

From a genetic point of view, the genus *Borrelia* can be classified into three monophyletic groups: the *Borrelia burgdorferi* sensu lato (Bb) complex, relapsing fever (RF) group and a third group of spirochetes associated with reptiles and echidna (*Tachyglossus aculeatus*) [3].

During the twentieth century in South America, *Borrelia venezuelensis* transmitted by *Ornithodoros rudis* and *Borrelia recurrentis* transmitted by the human louse were important pathogens in humans [4]. Wild mammals such as armadillos, monkeys and opossums were implicated as possible hosts [5–7] but never confirmed through molecular techniques.

Historically, the first report of spirochetes in opossums comes from Panama in 1931, with the animals showing an infection rate of 9.8% [7]. Subsequently, in 1946, Pifano detected spirochetes in thick blood films of opossums *Didelphis aurita* from Venezuela [8]. Although the vector of those spirochetes has yet to be confirmed, *O. puertoricensis*, a widely distributed tick in Central and northern South America, has been collected on *Didelphis virginiana* in Mexico [9]. Although *O. puertoricensis* remains to be confirmed as a vector of spirochetes, in Panama, Bermúdez et al. isolated a new RF group *Borrelia* (*B. puertoricensis*) from *O. puertoricensis* collected in burrows frequented by *Dasyprocta punctata* [10].

Despite opossums carry microorganisms of public health importance such as *Trypanosoma*, *Toxoplasma*, *Leishmania*, *Rickettsia* and *Leptospira* [11, 12], reports of *Borrelia* spp. in these animals are obscure and lack genetic confirmation. To elucidate whether opossums could carry *Borrelia* spirochetes, in this study we performed genetic screenings to detect *Borrelia* DNA in serum from opossums derived from a bank of samples in Colombia.

## Materials and methods

### Study area and capture of opossums

**Fig. 1:**
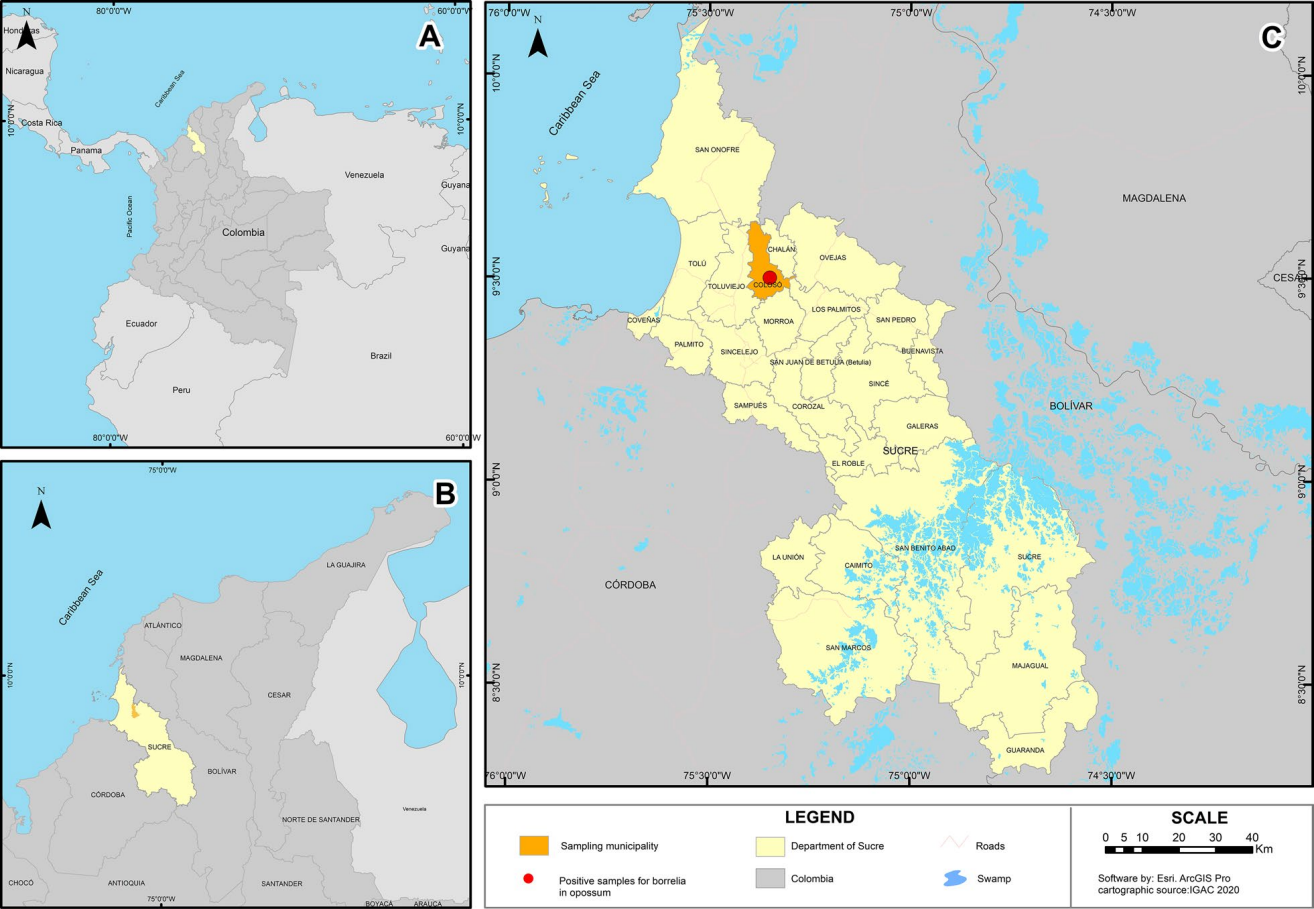
**A** Map of South America showing the location of the department of Sucre within Colombia. **B** Map of the Department of Sucre showing the municipality investigated. **C** Sampled municipality in the department of Sucre showing the opossum collection site

Four field trips were carried out on 5 days each in February, May and September 2017 and January 2018 in a rural area of the municipality of Colosó, department of Sucre (75°20′ 58.27″W–9°29′ 58.60″N) (Fig. 1). Ten Tomahawk-like traps baited with shells and chicken bones were set. Fifty-three captured opossums were identified as *Didelphis marsupialis*. Blood samples were collected in vacutainer tubes with EDTA after puncturing the caudal vein, and the animals were released into the wild. Serum was obtained through centrifugation. The capture of opossums was carried out with the permission of the National Authority for Environmental Licenses (ANLA, resolution no. 00914). Until use, serum samples were stored at the Instituto de Investigaciones Biológicas del Trópico of the University of Cordoba in Monteria, Northern Colombia.

### Molecular analyses

DNA extraction was performed on opossum sera using the GenJET Genomic DNA Purification kit (Thermo Scientific) following the manufacturer's instructions. A conventional PCR (cPCR) targeting the mammalian *ß-actin* gene was implemented as internal control for each extraction [13]. To detect *Borrelia* DNA, samples were subjected to real-time PCR (qPCR) targeting the *Borrelia* 16S rRNA gene as reported elsewhere [14]. Samples with cycle threshold values (Ct) ≤ 36 were considered positive [14]. Positive samples were then subjected to semi-nested PCR protocols to amplify longer fragments of the 16S rRNA and also the *flaB* gene [15, 16]. *Borrelia anserina* PL (DQ849625) genomic DNA was used as a positive control [17] and molecular grade water as a negative control. Amplicons of the expected size were Sanger sequenced at Macrogen (Seoul, Korea) (Table 1).

**Table 1:** Primers used to amplify *Borrelia* genes in this study

Gene	Round	Primer name	Secuencia 5′–3′	Tm [°C]	bp
16S rRNA qPCR	Screening	Bor16S3F	AGCCTTTAAAGCTTCGCTTGTAG	60	148
		Bor16S3R	GCCTCCCGTAGGAGTCTGG		
		probe Bor16S3P	[6FAM] CCGGCCTGAGAGGGTGAACGG		
16S rRNA	First round	FD3 [f]	AGAGTTTGATCCTGGCTTAG	54	1489
		T50 [r]	GTTACGACTTCACCCTCCT		
	Second round	FD3 [f]	AGAGTTTGATCCTGGCTTAG	56	730
		16 s-1 [r]	TAGAAGTTCGCCTTCGCCTCTG		
	Second round	16 s-2 [f]	TACAGGTGCTGCATGGTTGTCG	56	462
		T50 [r]	GTTACGACTTCACCCTCCT		
	Second round	Rec4 [f]	ATGCTAGAAACTGCATGA	54	520
		Rec9 [r]	TCGTCTGAGTCCCCATCT		
*flaB* (flagellin*)*	First round	FlaRL [f]	GCAATCATAGCCATTGCAGATTGT	55	665
		FlaLL [r]	ACATATTCAGATGCAGACAGAGGT		
	Second round	FLaRS [f]	CTTTGATCACTTATCATTCTAATAGC	55	491
		FlaLL [r]	ACATATTCAGATGCAGACAGAGGT		
	Second round	FlaRL [f]	GCAATCATAGCCATTGCAGATTGT	55	528
		FLaLS [r]	AACAGCTGAAGAGCTTGGAATG		

#### Short read sequencing

One sample positive for *Borrelia* detection was randomly selected to perform sequencing with the DNBSEQ-G50RS High-throughput (Rapid) technology (MGI, China). To this effect the MGIEasy FS DNA Prep kit (BGI, China) was employed according to the manufacturer's instructions. To obtain the opossum serum metagenome, shotgun sequencing was performed with a read length of 150 bp, paired end, with 2.97-Gb reads [18].

#### Bioinformatic analyses

Paired-end sequence reads were retrieved in fastq format and subjected to quality control. Low-quality sequences (Phred score < Q15), short reads (shorter than 15 bp) and adapter sequences were removed using fastp [19]. The quality of the reads was checked with FastQC [20]. Sequences corresponding to the host DNA (*D. marsupialis*) were removed by mapping the libraries against *Monodelphis domestica* (GCF_027887165.1) reference genome using Bowtie2. Notably, *M. domestica* is the sole species of the Didelphidae family with an available genome [21]. Unaligned reads were extracted with samtools [22] to perform a de novo metagenomic assembly with MEGAHIT using default parameters and a minimum contig length of 200 base pair (bp) [23]. The obtained contigs were then compared with BLASTn (using an E-value cutoff 10e^−3^) [24], and those aligned to the *Borrelia* genus were mapped against a multireference database consisting of representative *Borrelia* sequences (NC015921, NZCP028884, NZCP025785, NZCP036914, NZCP073148, NZA-YOT01000146, NZAYOU01000121, NZAZIT01000001, NC011244, NZLN609267, NZCP075379, NZCP073159, NC008710, NZCP073220) with Bowtie2. Gene annotation was done using Prokka [25] with a reference fasta (Genbank ID CP075379), minimum contig length of 200 bp and a *Borrelia* genus-specific database. Prodigal-metagenome option was used alongside Prokka to improve gene prediction. All the bioinformatic workflow was carried out in the Galaxy Project’s platform [26]. Sequences belonging to a multilocus sequence typing scheme commonly applied to *Borrelia* spp. (https://pubmlst.org) were selected for taxonomic assignment and building phylogenetic trees.

#### Phylogenetic analysis

Sequences generated by both Sanger and NGS were assembled in Ugene, and consensuses were compared against sequences reported in GenBank using BLASTn [24]. Alignments were built with Clustal Omega [27] with sequences downloaded from GenBank for each of the analyzed genes [28]. Aligned sequences were manually trimmed to match the query sequence lengths. Phylogenetic reconstructions were performed in IQtree with the maximum likelihood method; the best-fit nucleotide substitution model was obtained using ModelFinder [29]. The trees were reconstructed using 1000 bootstraps [30] and edited with iTOL v5 [31].

## Results

Amplicons of the expected size for the *ß-actin* gene were obtained in all the samples. Overall, 18.8% (10/53) were positive for *Borrelia* spp. 16S rRNA gene by qPCR with Ct ranging between 23 and 33. Of those positive samples, 80% (8/10) and 60% (6/10) were positive for the 16S rRNA and *flaB* genes by semi-nested PCR, respectively. The sizes of the obtained amplicons were 1112–1474 bp for the 16S rRNA gene and 627–636 bp for the *flaB* gene (Table 1). Twenty-two gene segments with sizes ranging between 218–1460 bp of 81.82–100% of identity with *B. puertoricensis* were retrieved from the sole sample submitted to NGS sequencing (see Additional file 1: Table S1, S2). The sequences were deposited in GenBank with the accession numbers OQ944473–OQ944479, OQ725656–OQ725662 and OQ871584.

**Fig. 2:**
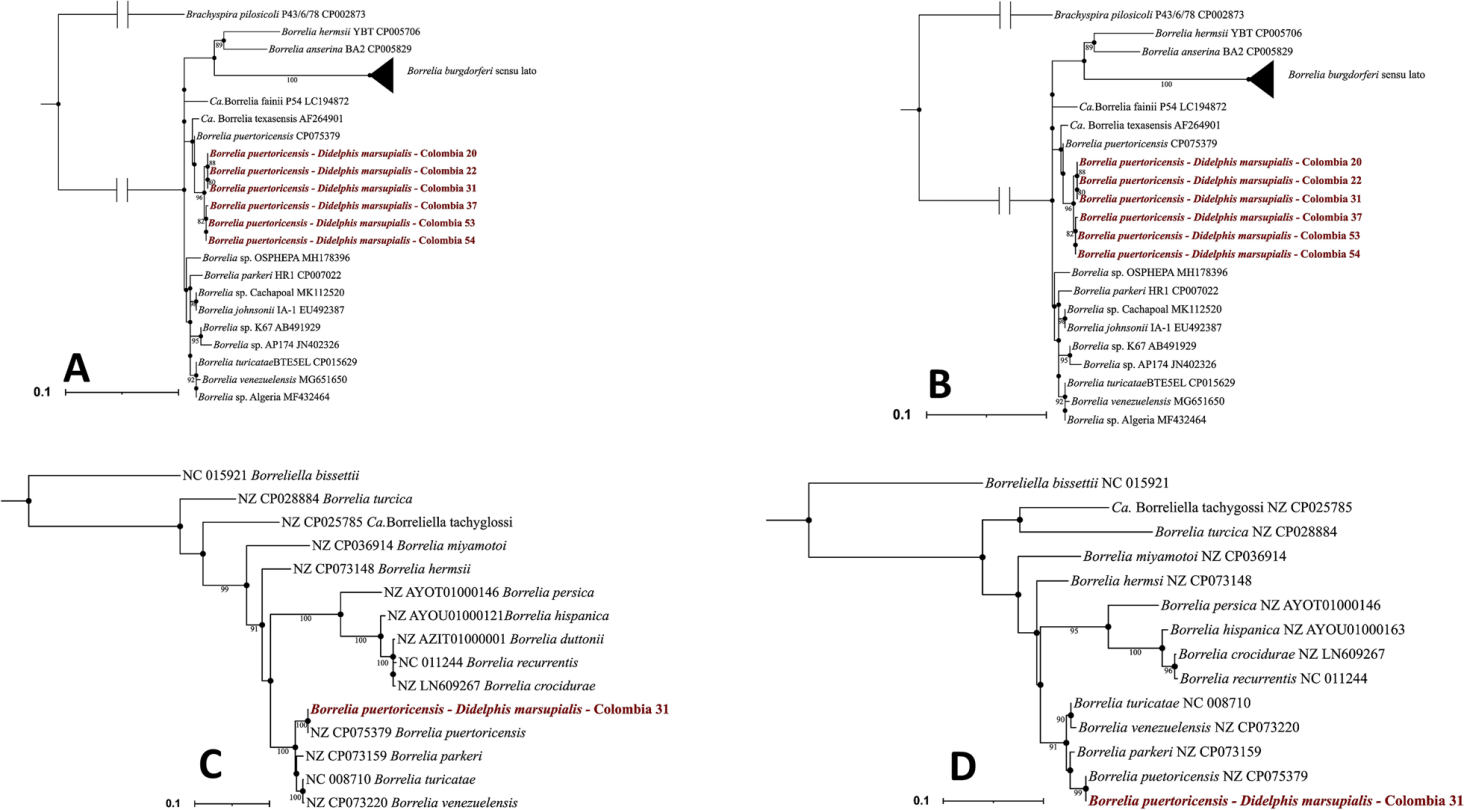
**A** Phylogenetic tree of the 16S rRNA gene built with 78 sequences (71 downloaded from Genbank and 6 obtained in the present study). **B** Phylogenetic tree of the *flaB* gene built with 39 sequences (33 downloaded from Genbank and obtained in the present study). These two trees were rooted with *Brachyspira pilosicoli*. **C** Phylogenetic tree of the *pyrG* gene built with 14 sequences (13 downloaded from Genbank 1 obtained in the present study). **D** Phylogenetic tree of the *rplB* gene built with 14 sequences (13 downloaded from Genbank 1 obtained in the present study). These two trees were rooted with *Borrelia bissetti*. The sequences generated this study are found in the tree colored in red. The trees were built using the TPM3 + F + I + G4 for the 16S rRNA and flaB gene, TR + F + G4 for the pyrG gene and K3PU + F + G4 for the rplB model as selected based on BIC (16S rRNA = 10,831.821, *flaB* = 7940.507 *pyrG* = 14,184.493, *rplB* = 6816.236)

Of the genes retrieved by the bioinformatic analyses, the 50S ribosomal protein L2 gene (*rplB*) and the CTP synthase (*pyrG*) were used in the taxonomic assignment and phylogenetic analysis since they belong to a multilocus sequence typing scheme of the genus *Borrelia* [32]. BLASTn comparisons performed to the 16S rRNA*, flaB, rplB* and *pyrG* gene sequences showed 99.2–100% identity with *B*. *puertoricensis* isolated from *O. puertoricensis* of Panama [10]. For the phylogenetic reconstructions, 78, 39, 14 and 14 sequences were downloaded for the alignments of the 16S rRNA, *flaB*, *rplB* and *pyrG* genes, respectively. All four phylogenies depicted a logical topology grouping the species into the *B. burgdorferi* sensu lato group, transitional group and FR group. The sequences of *Borrelia* retrieved in this study clustered into a monophyletic clade with *B. puertoricensis* with branch support ranging between 82 and 100% (Fig. 2).

## Discussion

Phylogenetic analyses performed in this study using the 16S rRNA, *flaB*, *rplB* and *pyrG* gene demonstrated that the detected species correspond to *B*. *puertoricensis*, which was recently isolated from the tick *O*. *puertoricensis* collected from burrows frequented by *D. punctata* in Panama [10]. In the study of Bermudez et al., the taxonomic position of *B*. *puertoricensis* was evaluated by BLASTn comparisons and by concatenating four loci (*IGS*, *rrs*, *flaB* and *gyrB*) to perform phylogenetic analyses, which collectively showed that the isolated spirochete was closely related with *Borrelia* *turicatae* and *B*. *parkeri* [10]. Although in our study the gene sequences of the phylogenies were not exactly the same, our results agree with those obtained by Bermúdez et al. in that they conserve the same topology despite evaluating different genes independently (Fig. 2).

This study provides the first molecular characterization of a *Borrelia* sp. in opossums. However, the first evidence of opossums as potential hosts of *Borrelia* came with the observation of spirochetes in blood of *D. marsupialis* in Panama in 1931 [7]. At that time, 61 opossums were screened and 6 (9.8%) were positive [7]. Later, in 1946, Pifano detected spirochetes in thick blood smears of *D. aurita* in Venezuela, and he attributed the species to “*Spirocheta venezuelensis*” [8], which is currently recognized as a synonym of *B. venezuelensis*. These previous reports were based only on morphology and corresponded to the sole evidence of *Borrelia* in opossums along the American continent.

Although spirochetes of genus *Borrelia* have been observed and now genetically identified as *B. puertoricensis*, at least in one opossum of this study, the vector remains unknown. However, laboratory experiments show that *O. puertoricensis* transmits *B. puertoricensis* to mice [10]. Interestingly, Ballados-González et al. collected *O*. *puertoricensis* in opossums (*D. virginiana*) from Mexico [9], a fact that suggests that this soft tick species could be the vector of *B*. *puertoricensis* to opossums. However, in Colombia, soft ticks parasitizing opossums have not been collected.

Given that in our study *B. puertoricensis* was detected in serum of the opossums, these mammals could be involved as reservoir hosts for these microorganisms; however, more studies are needed to confirm this hypothesis. Opossums have synanthropic habits and spread pathogens in nature [11, 12]. Indeed, these animals are important in the transmission cycles of zoonotic diseases such as trypanosomiasis, toxoplasmosis, leishmaniasis, rickettsiosis and leptospirosis [11, 12]. Therefore, it would be important to know the epidemiological role that opossums may play in the transmission cycles of RF *Borrelia* and to further elucidate the vector of the spirochetes (e.g., *Ornithodoros* ticks).

This study demonstrates the presence RF group *B. puertoricensis* in opossums from Colombia through the sequencing of four genes of the spirochete from serum of infected animals. In Colombia, the study of RF group spirochetes dates from the first half of the twentieth century [4]. Our results highlight that RF group spirochetes of genus *Borrelia* do circulate in wild animals, and attention should be paid to opossums as potential reservoirs.

### Supplementary Information

**Additional file 1: Table S1.** *Borrelia puertoricensis* annotated genes. **Table S2.** Results of qPCR for of *Borrelia*-positive samples, 16S rRNA and *flaB* PCR.

## Data Availability

Sequences are available in GenBank with accession numbers OQ944473–OQ944479, OQ725656–OQ725662 and OQ871584.

## Abbreviations

Bb: *Borrelia burgdorferi* sensu lato
RF: Borreliae of the relapsing fever
