# Supplementary material for: Borrelia puertoricensis in opossums (Didelphis marsupialis) from Colombia
Source: Parasit Vectors. 2023 Dec 4;16:448. doi: 10.1186/s13071-023-06016-4 (PMC10694944; doi:10.1186/s13071-023-06016-4)
Supplement: Supplementary file 1 — Additional file 1: Table S1. Borrelia puertoricensis annotated genes. Table S2. Results of qPCR for of Borrelia-positive samples, 16S rRNA and flaB PCR. [file 13071_2023_6016_MOESM1_ESM.docx]

Table S1*. Borrelia puertoricensis* annotated gene features.

| **ID** | **Type of sequencing** | **Genome location** |  | **Contig length** | **Protein** | **Identity** | **Access number Genbank** |
| --- | --- | --- | --- | --- | --- | --- | --- |
| 31 | Nanoball secuencing BGI | 924300 | 924869 | 569 | *hypothetical protein* | 98.95% | OQ871584 |
|  |  | 4715 | 5716 | 1001 | *hypothetical protein* | 95.21% |  |
|  |  | 921008 | 921949 | 941 | *Tryptophan--tRNA ligase* | 98.41% |  |
|  |  | 70411 | 70821 | 410 | *Peptide chain release factor RF2* | 99.27% |  |
|  |  | 82129 | 82803 | 674 | *Competence protein ComM* | 99.56% |  |
|  |  | 120051 | 120275 | 224 | *Elongation factor Ts* | 97.33% |  |
|  |  | 120296 | 120598 | 302 | *hypothetical protein* | 99.01% |  |
|  |  | 141572 | 142042 | 470 | *Swarming motility protein SwrC* | 99.36% |  |
|  |  | 149685 | 150350 | 665 | *hypothetical protein* | 90.99% |  |
|  |  | 170045 | 170263 | 218 | *Translation initiation factor IF-1* | 98.63% |  |
|  |  | 238066 | 238593 | 527 | *Obg-like ATPase 1* | 98.86% |  |
|  |  | 272689 | 273696 | 1007 | *Membrane-bound lytic murein transglycosylase* | 99.70% |  |
|  |  | 281237 | 281365 | 128 | *hypothetical protein* | 100.00% |  |
|  |  | 281453 | 281761 | 308 | *hypothetical protein* | 98.06% |  |
|  |  | 281789 | 282154 | 365 | *hypothetical protein* | 99.18% |  |
|  |  | 347441 | 347791 | 350 | *Enolase* | 97.44% |  |
|  |  | 367507 | 367938 | 431 | *RNA polymerase-binding transcription factor CarD* | 100.00% |  |
|  |  | 371228 | 371779 | 551 | *Flagellum site-determining protein YlxH* | 92.93% |  |
|  |  | 391756 | 392460 | 704 | *Periplasmic trehalase* | 96.17% |  |
|  |  | 494844 | 495254 | 410 | *2',3'-cyclic-nucleotide 2'-phosphodiesterase"* | 100.00% |  |
|  |  | 414233 | 414703 | 470 | *hypothetical protein* | 98.09% |  |
|  |  | 419781 | 420266 | 485 | *hypothetical protein* | 99.38% |  |
|  |  | 433602 | 434252 | 650 | *Sensor histidine kinase RcsC* | 94.47% |  |
|  |  | 450658 | 450939 | 281 | *hypothetical protein* | 90.43% |  |
|  |  | 499634 | 500251 | 617 | *Elongation factor Tu* | 100.00% |  |
|  |  | 577251 | 577985 | 734 | *hypothetical protein* | 86.99% |  |
|  |  | 582920 | 583075 | 155 | *hypothetical protein* | 100.00% |  |
|  |  | 582643 | 583023 | 380 | *hypothetical protein* | 99.21% |  |
|  |  | 597768 | 598271 | 503 | *CTP synthase* | 99.41% |  |
|  |  | 639425 | 639913 | 488 | *Chemoreceptor glutamine deamidase CheD* | 100.00% |  |
|  |  | 643026 | 643430 | 404 | *Trigger factor* | 100.00% |  |
|  |  | 689050 | 689511 | 461 | *PTS system glucoside-specific EIICBA component"* | 81.82% |  |
|  |  | 766727 | 767062 | 335 | *hypothetical protein* | 99.11% |  |
|  |  | 916230 | 916775 | 545 | *hypothetical protein* | 98.90% |  |
|  |  | 502246 | 502842 | 528 | *50S ribosomal protein L23* | 100.00% | OQ944479 |
| 20 | Sanger | 148172 | 148801 | 629 | *flagellin* | 99.53% | OQ944473 |
| 22 |  | 148167 | 148803 | 636 | *flagellin* | 99.53% | OQ944474 |
| 31 |  | 148167 | 148803 | 636 | *flagellin* | 99.53% | OQ944475 |
| 37 |  | 148169 | 148804 | 635 | *flagellin* | 99.22% | OQ944476 |
| 53 |  | 148168 | 148804 | 636 | *flagellin* | 99.37% | OQ944477 |
| 54 |  | 148176 | 148803 | 627 | *flagellin* | 99.52% | OQ944478 |
| 14 |  | 446321 | 447781 | 1460 | *16rRNA* | 99.93% | OQ725656 |
| 20 |  | 446321 | 447780 | 1459 | *16rRNA* | 99.93% | OQ725657 |
| 22 |  | 446321 | 447433 | 1112 | *16rRNA* | 99.91% | OQ725658 |
| 31 |  | 446314 | 447786 | 1472 | *16rRNA* | 99.93% | OQ725659 |
| 32 |  | 446328 | 447783 | 1455 | *16rRNA* | 100.00% | OQ725660 |
| 53 |  | 446322 | 447796 | 1474 | *16rRNA* | 99.93% | OQ725661 |
| 54 |  | 446321 | 447786 | 1465 | *16rRNA* | 99.93% | OQ725662 |

**Table S2***. Didelphis marsupialis* positive qPCR for *Borrelia* spp. and 16S rRNA and *flaB* gene results.

| **Possums positive by qPCR** | **16S rRNA gene cPCR results** | ***flaB* gene cPCR results** |
| --- | --- | --- |
| 8 | Positive | Negative |
| 14 | Positive | Negative |
| 20 | Positive | Positive |
| 22 | Positive | Positive |
| 31 | Positive | Positive |
| 32 | Positive | Negative |
| 37 | Positive | Positive |
| 40 | Negative | Negative |
| 53 | Positive | Positive |
| 54 | Positive | Positive |
|  |  |  |
